# Immunomodulatory Effects of Genetic Alterations Affecting the Kynurenine Pathway

**DOI:** 10.3389/fimmu.2019.02570

**Published:** 2019-11-06

**Authors:** Fanni A. Boros, László Vécsei

**Affiliations:** ^1^Department of Neurology, Faculty of Medicine, Albert Szent-Györgyi Clinical Center, University of Szeged, Szeged, Hungary; ^2^MTA-SZTE Neuroscience Research Group of the Hungarian Academy of Sciences, University of Szeged, Szeged, Hungary; ^3^Department of Neurology, Interdisciplinary Excellence Centre, University of Szeged, Szeged, Hungary

**Keywords:** kynurenine pathway, IDO, TDO, KMO, immunomodulation, genetic manipulation

## Abstract

Several enzymes and metabolites of the kynurenine pathway (KP) have immunomodulatory effects. Modulation of the activities and levels of these molecules might be of particular importance under disease conditions when the amelioration of overreacting immune responses is desired. Results obtained by the use of animal and tissue culture models indicate that by eliminating or decreasing activities of key enzymes of the KP, a beneficial shift in disease outcome can be attained. This review summarizes experimental data of models in which IDO, TDO, or KMO activity modulation was achieved by interventions affecting enzyme production at a genomic level. Elimination of IDO activity was found to improve the outcome of sepsis, certain viral infections, chronic inflammation linked to diabetes, obesity, aorta aneurysm formation, and in anti-tumoral processes. Similarly, lack of TDO activity was advantageous in the case of anti-tumoral immunity, while KMO inhibition was found to be beneficial against microorganisms and in the combat against tumors, as well. On the other hand, the complex interplay among KP metabolites and immune function in some cases requires an increase in a particular enzyme activity for the desired immune response modulation, as was shown by the exacerbation of liver fibrosis due to the elimination of IDO activity and the detrimental effects of TDO inhibition in a mouse model of autoimmune gastritis. The relevance of these studies concerning possible human applications are discussed and highlighted. Finally, a brief overview is presented on naturally occurring genetic variants affecting immune functions *via* modulation of KP enzyme activity.

## Introduction

The kynurenine pathway (KP) is the main route of Trp metabolism. The enzymes of the pathway generate numerous metabolites, some of which are pro-inflammatory and/or generate free radicals, while others are known to be anti-inflammatory and/or scavenge free-radicals. Strong links between KP function and the immune system are demonstrated by extensive amounts of data on changes in the levels of KP metabolites and enzyme activities in diseases accompanied by alterations in immune function. Also, inflammatory cytokines are known to enhance the expression of a key KP enzyme, indoleamine 2,3-dioxygenase (IDO). Imbalances in the pathway can be detrimental, as excessive production of either pro-, or anti-inflammatory metabolites can contribute to the development of autoimmunity and/or lead to inefficient immune response against pathogens. Therefore, the understanding of how the KP changes in different immunological states, and, the reverse, how KP effects immunological responses, is cardinal both for understanding the true nature of specific diseases and for identification of therapeutic targets. Genetic manipulations leading either to enhancement or inhibition of the expression of KP enzymes might be a feasible way of restoring the imbalance of the pathway in various diseases. Naturally occurring genetic variations in the coding regions in several genes coding for KP enzymes have been identified [for a review see (1)]. In the majority of these, however, a causal relation between a specific gene variant and disease development has not been elucidated.

This review summarizes available data on the effects of expression modification of KP enzyme coding genes with specific attention to immune modulation. Following a brief overview of the metabolites and enzymes of the pathway, we summarize observations which indicate links between KP and immune function. This is followed by an overview of findings obtained by the use of models with targeted ablation and up- or down-regulation of KP enzymes. With respect to diseases related to disorders of the immune system, such as infectious diseases, chronic inflammation, autoimmunity and cancer, these models have focused on three KP enzymes: IDO, tryptophan 2,3-dioxygenase (TDO) and kynurenine 3-monooxygenase (KMO) (Tables 1–5). These enzymes and, in particular, IDO are also targeted by several pharmacochemical interventions. Discussion of that field is out of the scope of this review, as we focus on gene level interventions. Readers interested in pharmacologic interventions of KP enzymes can find excellent summaries of the field in Ye et al. (45) and Lemos et al. (46). In the final section, we provide a summary of available data on those naturally occurring KP gene variants which are believed to be associated with different human diseases affecting immune function.

**Table 1:** Effects of modulation of IDO function by genetic manipulation in *in vivo* and *in vitro* models of systemic inflammation, viral, and bacterial infections.

**Gene**	**Type of genetic modulation**	**Disease modeled**	**Study design**	**Effect of gene modulation**	**References**
*IDO*	IDO^−/−^	Systemic inflammation	Mouse model of LPS induced sepsis	Restoration of imbalance of pro-and anti-inflammatory cytokines, increased survival rate	(2)
	IDO^−/−^	Viral infection	Murine leukemia virus induced murine AIDS model	Decreased virus replication; increased number of pDCs and increased type I IFN production; increased survival rate following *Toxoplasma gondii* infection	(3)
	IDO^−/−^	Viral infection	ECMV induced mouse model of acute viral myocarditis	Decreased virus replication and myocardium necrosis; higher survival rate	(4)
	IDO^−/−^	Pain hypersensitivity related to viral infection	Pain hypersensitivity induced by Influenza A virus and MuLV infection	Diminished acute and chronic pain sensitivity related to influenza A and MuLV infections, respectively	(5)
	IDO overexpression	Viral infection	HeLa cells transfected with pcDNA3-IDO	Overexpression of IDO prior to viral infection diminished viral replication thus decreasing infection spread to the neighboring cells	(6)
	IDO^−/−^	Viral infection	LP-BPM5 retrovirus infection of mice - a model of murine AIDS	Gene knockout did not have any effect on disease progression and viral load	(7)
	IDO^−/−^	Bacterial infection	Mouse model of *Mycobacterium tuberculosis* infection	*In vitro* findings showed enhanced T cell proliferation after infection, however, *in vivo* no significant difference could be observed in survival rate or in the number of activated T cells	(8)
	IDO^−/−^	Bacterial infection	Murine cystitis model provoked by uropathogen *Escherichia coli* infection	Increased levels of pro-inflammatory cytokines, higher granulocyte accumulation, and local inflammation of the bladder and decreased survival of the extracellular bacteria	(9)
	IDO^−/−^	Bacterial infection	Mouse model of *Rhodococcus equi* infection	Decreased levels of TGFβ and FOXP3 expression in the liver tissue indicating reduced T regulatory cell responses and prolonged liver inflammation	(10)

*IDO, Indoleamine 2,3-dioxygenase gene; IDO^−/−^, IDO knockout; pDC, plasmocytoid dendritic cell; IFN, interferon; ECMV, encephalomyocarditis virus; MuLV, murine leukemia virus; TGFβ, transforming growth factor beta; FOXP3, forkhead box P3*.

**Table 2:** Effects of modulation of IDO function by genetic manipulation in animal models of allergy and autoimmunity.

**Gene**	**Type of genetic modulation**	**Disease modeled**	**Study design**	**Effect of gene modulation**	**References**
*IDO*	IDO^−/−^	Airway allergy	Mouse model of acute and chronic allergic airway inflammation	Decrease in Th2 response upon exposure to allergen: diminished Th2 cell activation, Th2 cytokine production, decreased airway inflammation, mucus secretion, and airway hyperresponsiveness	(11)
	IDO^−/−^	Autoimmunity	Mice models of sJIA, MAS and sHLH	No difference in the symptoms of IDO^−/−^ animals compared to WT—possibility of the presence of other Trp metabolizing enzymes restoring the absence of Ido	(12)
	IDO^−/−^	Autoimmunity	EAE mouse model of MS	Immunization with MOG, systemic treatment with DNPs or c-diGMP induced STING signaling, thus potent regulatory immune responses could be achieved, leading to restrained EAE severity and delayed disease onset. However, in the case of lack of *IDO* in hematopoietic cells, no therapeutic response could be observed	(13)
	IDO^−/−^	Autoimmunity	EAE mouse model of MS	Exacerbated EAE disease severity, increased encephalitogenic Th1 and Th17 responses and diminished Treg responses in IDO^−/−^ animals.	(14)
	IDO^−/−^	Autoimmunity	CIA mouse model of RA	More severe disease demonstrated by increased erosion and cellular infiltration of the joints of IDO^−/−^ animals, higher production of IFNγ and IL-17 in the lymph nodes and higher Th1 and Th17 cell frequency in paws	(15)
	AdIDO	Autoimmunity	CIA rat model of RA	Significant reduction of bony destruction, soft tissue swelling and synovial hyperplasia, indicating decreased disease severity	(16)
	Transfection with *Ido*	Autoimmunity	NOD mouse model of T1D	After TGFβ treatment production of pro-inflammatory cytokines (IL-6 and TNFα) was decreased and pancreatic β-cell auto-antigen generation was diminished	(17)
	IDO^−/−^	Autoimmunity	MRLlpr/lpr mouse model of SLE	The injection of apoptotic thymocytes in IDO^−/−^ MRLlpr/lpr animals caused elevation of autoantibody titers, pro-inflammatory cytokine production and dysregulated T cell responses leading to lethal autoimmunity due to renal failure	(18)

*IDO, Indoleamine 2,3-dioxygenase gene; IDO^−/−^, IDO knockout; sJIA, systemic juvenile idiopathic arthritis; MAS, macrophage activation syndrome; sHLH, secondary hemophagocytic-lymphohistiocytosis; WT, wild type; Trp, tryptophan; EAE, experimental autoimmune encephalitis; MS, multiple sclerosis; MOG, myelin oligodendrocyte glycoprotein; DNP, DNA nanoparticle; c-diGMP, cyclic diguanylate monophosphate; CIA, collagen induced arthritis; RA, rheumatoid arthritis; IFNγ, interferon gamma; AdIDO, adenoviral vector-mediated IDO gene delivery; NOD, non-obese diabetic; T1D, type 1 diabetes; TGFβ, transforming growth factor beta; MRLlpr/lpr, Lupus-prone Murphy Roths large mice; SLE, systemic lupus erythematosus*.

**Table 3:** Effects of modulation of IDO function by genetic manipulation in transplant animal models.

**Gene**	**Type of genetic modulation**	**Disease modeled**	**Study design**	**Effect of gene modulation**	**References**
*IDO*	Adenoviral *Ido* gene transfer	Transplantation	Adenoviral gene transfer into pancreatic islets; transplantation into diabetogenic mice	Prolonged survival of transplanted tissue; depletion of local Trp; inhibition of T cell proliferation	(19)
	EIAV based *Ido* gene transfer	Transplantation	Mouse model of corneal transplant	Prevention of allogeneic T cell responses; prolonged corneal graft survival	(20)
	hIDO gene transfer *via* PEI	Transplantation	Rat model of lung transplant	Blockage of local T cell responses, inhibition of intracellular ROS formation, thus reducing necrosis and apoptosis of lung cells	(21)
	hIDO gene transfer *via* PEI	Transplantation	Rat model of lung transplant	Selective decrease of complex I activity of the electron transport chain, leading to decreased ATP production in the lung infiltrating T cells, causing damage in their cytotoxic properties	(22)
	Sleeping beauty transposon mediated hIDO delivery	Transplantation	Rat model of lung transplant, investigation of lung fibrosis	Diminished collagen deposition in IDO^+/+^ lungs, resulting in a more preserved bronchus-alveolar architecture. *In vitro* findings revealed that IDO^+/+^ lung cells inhibited the TGFβ mediated proliferation of fibroblasts	(23)
	adenoviral *Ido* gene transfer	Transplantation	Rat model of skin transplant	Wounds with IDO expressing fibroblast healed faster than those with IDO^−/−^ fibroblasts due to enhanced capillary formation	(24)
	adenoviral *Ido* gene transfer	Transplantation	Rat model of cardiac allograft survival	Decreased infiltration of the cardiac allograft with monocytes, macrophages and T cells, accompanied by diminished intragraft levels of IFNγ, TNFα, TGFβ, IL-1β, resulting in prolonged graft survival	(25)
	PEI carrier hIDO transfer	Transplantation	Mouse model of lung transplantation	Prolonged graft survival due to inhibited early T cell responses and diminished memory T cell formation. T cell inhibiting properties were found to be due to the impairment of calcium signaling of the cells	(26)

*IDO, Indoleamine 2,3-dioxygenase gene; IDO^−/−^, IDO knockout; Trp, tryptophan; EIAV, Equine infectious anemia virus; PEI, polimer polyethilenimine; ROS, reactive oxygen species; TGFβ, transforming growth factor beta; IFNγ, interferon gamma; TNFα, tumor necrosis factor alpha*.

**Table 4:** Effects of modulation of IDO function by genetic manipulation in *in vitro* and *in vivo* models of chronic inflammation and cancer.

**Gene**	**Type of genetic modulation**	**Disease modeled**	**Study design**	**Effect of gene modulation**	**References**
*IDO*	IDO^−/−^	Chronic inflammation	Mouse model of DR	Reduced retinal capillary degeneration	(27)
	IDO^−/−^	Chronic inflammation	AngII induced atherosclerosis mouse model	Reduced ROS production; diminished endothelial cell dysfunction and apoptosis	(28)
	IDO^−/−^	Chronic inflammation	Mouse model of AAA: *Ldlr*^−/−^ mice infused with AngII and fed with HFD	Reduced VSMC apoptosis	(29)
	IDO^−/−^ and siRNA mediated *Kynu* silencing	Chronic inflammation	AngII induced AAA formation in *ApoE*^−/−^ mice	Protection against AAA formation—decrease in elastic lamina degradation and aortic expansion	(30)
	IDO^−/−^	Chronic inflammation	Mouse model of obesity	Lower body weight and fat mass; increased number of M2 (anti-inflammatory) macrophages in the WAT; protection against the development of liver steatosis and insulin resistance; diminished LPS plasma levels	(31)
	IDO^−/−^	Chronic inflammation	CCl4 induced mouse model of hepatic fibrosis	Aggravation of liver fibrosis: higher TNFα producing macrophages in the liver; higher TNFα and fibrogenic factor expression	(32)
	IDO^−/−^	Intestinal immunity	*Citrobacter rodentium*-induced colitis mouse model	Attenuated intestinal inflammatory response: less edema, cellular infiltration, epithelial damage and reduced intestinal colonization of bacteria	(33)
	*IDO* silencing *via* siRNA	Tumor immunity	B16F10 melanoma cells *in vitro* and mouse model	Decrease in tumor size; prevention of T cell apoptosis; restoration of host antitumor immunity	(34)
	*IDO* silencing *via* shIDO-ST	Tumor immunity	B16F10 melanoma mouse model	Tumor growth is attenuated and the number of lung metastases was diminished	(35)
	IDO silencing *via* shRNA	Tumor immunity	SKOV-3 human ovarian cancer cell line and mouse model	Decrease in tumor growth, peritoneal dissemination and ascites formation, increase in the number of tumor infiltrating NK cells *in vivo;* increased sensitivity to NK cells *in vitro*	(36)
	IDO silencing *via* shRNA	Tumor immunity	Genetic downregulation of IDO in A549 human lung adenocarcinoma cells	Enhanced sensitivity of cells to FK866, MX, pemetrexed and gemcitabine therapy	(37)

*IDO, Indoleamine 2,3-dioxygenase gene; Ido^−/−^, Ido knockout; Kynu, kynureninase gene; siRNA, small interfering RNA; shIDO-ST, shRNA: short hairpin RNA; DR, diabetic retinopathy; AngII, Angiotensin II; AAA, abdominal aortic aneurysm; Ldlr^−/−^, low density lipoprotein—receptor deficient; HFD, high fat diet; ApoE^−/−^, Apolipoprotein E knockout; CCl4, carbon-tetrachloride; ROS, reactive oxygen species; VSMC, vascular smooth cell; WAT, white adipose tissue; LPS, lipopolysaccharide; TNFα, tumor necrosis factor alpha; NK cell, natural killer cell; MX, methoxyamine*.

**Table 5:** Effects of modulation of TDO and KMO function by genetic manipulation in *in vitro* and *in vivo* models.

**Gene**	**Type of genetic modulation**	**Disease modeled**	**Study design**	**Effect of gene modulation**	**References**
*TDO*	Lack of *Tdo* expression	Tumor immunity	P815 mouse tumor model	Slower tumor progression, higher number of cytolytic T cells in the tumor microenvironment	(38)
	TDO^−/−^	Autoimmunity	EAE mouse model of MS	Protective effects against neuronal loss in the spinal cord	(39)
	TDO expression	Infection	HeLa T-Rex cells transfected with pcDNA4-*Tdo* vector containing human liver *TDO* cDNA	Antiparasitic, antiviral, and antibacterial effect; suppression of T cell proliferation	(40)
*KMO*	KMO^−/−^	Viral infection	EMCV induced mouse model of viral myocarditis	Higher survival rate of *Kmo*^−/−^ animals; decrease in the cellular infiltration of marophages and neutrophiles in heart tissue	(41)
	siRNA mediated *Kmo* silencing	Autoimmunity	Mouse model of autoimmune gastritis	Disease exacerbation due to excessive Th17 cell formation	(42)
	KMO^−/−^	Chronic inflammation	Diabetic mouse and zebrafish models	Proteinuria related to the malfunctioning of kidney podocytes (proposedly due to NAD^+^ depletion)	(43)
	KMO^−/−^	IRI	IRI leading to AKI in a mouse model	Decreased renal tubular necrosis and neutrophil granulocyte infiltration	(44)

*TDO, Tryptophan 2,3-dioxygenase gene; TDO-/-, TDO knockout; KMO, Kynurenine 3-monooxygenase gene; KMO-/-, KMO knockout; EAE, experimental autoimmune encephalitis; MS, multiple sclerosis; EMCV, encephalomyocarditis virus; IRI, ischemia-reperfusion injury; AKI, acute kidney injury*.

## Kynurenine Pathway—The Main Route of Tryptophan Metabolism

**Figure 1:**
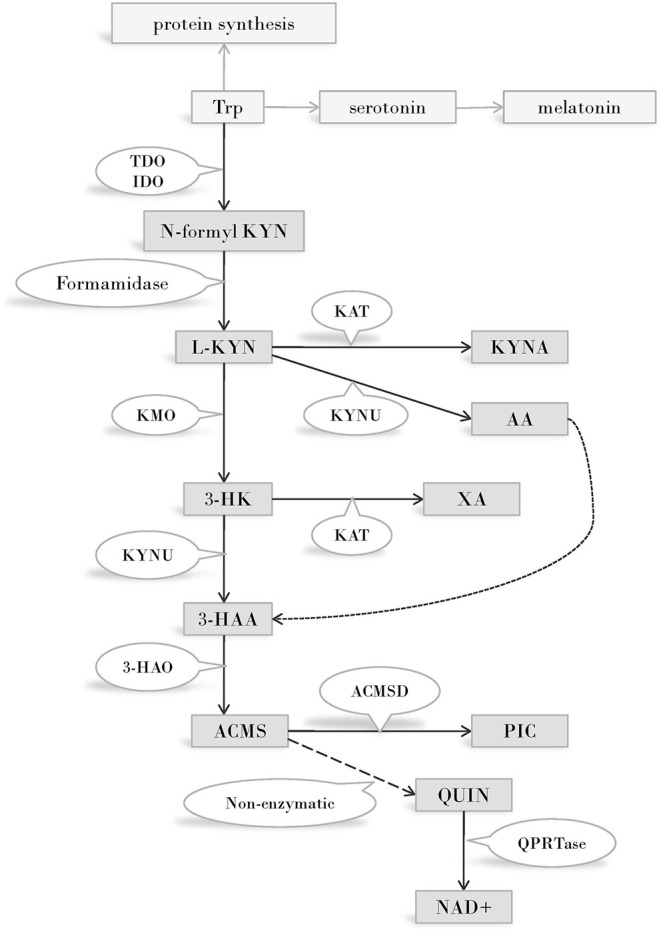
The kynurenine pathway of tryptophan metabolism. Enzymes of the KP metabolise Trp into products possessing immune- and neuromodulatory properties. By the utilization of Trp and generation of NAD coenzyme precursor the KP has profound effects on cellular protein and energy metabolism. Several internal metabolites of the pathway play role on redox regulation and have neuroprotective - or neurotoxic effects. Immune functions are modified by the KP both directly, via immuno modulatory metabolites and indirectly, via changing the metabolism of immune cells by altering amino acid availability, redox status and energy balance. Abbreviations: Trp: tryptophan; TDO:tryptophan 2,3-dioxygenase; IDO:indoleamine 2,3-dioxygenase; N-formyl KYN:N-formyl-kynurenine; L-KYN:L-kynurenine; KAT:kynurenine aminotransferase; KYNA: kynurenic acid; KYNU:kynureninase; AA:anthranilic acid; KMO:kynurenine 3-monooxygenase; 3-HK:3-hydroxy kynurenine; XA: xanthurenic acid; KYNU:kynureninase; 3-HAA: 3-hydroxyanthranilic acid; 3-HAO:3-hydroxyanthranilate 3,4-dioxygenase; ACMS: 2-amino-3-carboxymuconate-semialdehyde; ACMSD: aminocarboxymuconate-semialdehyde-decarboxylase; PIC: picolinic acid; QUIN: quinolinic acid; QPRTase:quinolinate phosphoribosyltransferase; NAD^+^: nicotinamide adenine dinucleotide; CNS: central nervous system.

Disregarding protein synthesis, the KP is the main route of Trp metabolism, both in the peripheral and in the central nervous system (CNS) (Figure 1). In the CNS, 95 percent of the resident Trp is metabolized *via* the KP, and only the minority of the amino acid is transformed into serotonin and melatonin. In consecutive steps of the pathway, numerous metabolites possessing immune- and neuromodulatory properties are synthesized (47).

The first and rate limiting step of Trp metabolism is the conversion of the amino acid into N-formyl-L-kynurenine. This step is catalyzed by one of three enzymes: IDO (often referred to as IDO1), IDO2, or TDO. (Prior to the discovery of IDO 2, “IDO” designation was used exclusively. Today IDO and IDO1 are used as synonyms and IDO2 is reserved for the enzyme recognized in 2007. In this review we will use IDO unless we are referring to IDO2). TDO is expressed mainly in the liver, thus plays a cardinal role in regulating the amount of available Trp throughout the body, outside the CNS. IDO is expressed in several human tissues, among them various cell types of the immune system (48). The enzyme plays a key role in reactions leading to the synthesis of immunoactive KP metabolites, consequently its role in immunomodulation is expected. IDO2 expression pattern and function is not known in detail. A strong argument against the role of this enzyme in Trp metabolism is the frequent occurrence of an IDO2 gene variant that gives rise to a non-functioning enzyme (49), and the high Michaelis Constant of the enzyme for Trp, which is 100-fold above the physiological concentrations of the amino acid (50).

N-formyl-L-kynurenine is converted to L-kynurenine (L-KYN) by formamidase. L-KYN is an important branch point of the KP as it can be alternatively metabolized into three different metabolites of which some are neurotoxic, while others possess neuroprotective and antioxidant properties (51, 52). Firstly, L-KYN can be metabolized into kynurenic acid (KYNA) by kynurenine aminotransferases (KATI-IV) from which KATII plays the most important role in the human CNS (53, 54). Secondly, L-KYN is also a substrate of kynureninase (KYNU), an enzyme responsible for the formation of anthranilic acid (AA). Finally, the third route of L-KYN metabolism is catalyzed by KMO to form 3-hydroxy kynurenine (3-HK) which can be further transformed into xanthurenic acid (XA) by KATs. 3-HK and AA can both be metabolized into 3-hydroxyanthranilic acid (3-HAA), which, alongside with 3-HK, have free-radical generating properties, thus can lead to oxidative stress and neurodegeneration (55). However, depending on the redox properties of the cell, 3-HK and 3-HAA can also serve as antioxidant molecules (56).

Further down the pathway, the unstable 2-amino-3-carboxymuconate-semialdehyde (ACMS) is formed by 3-hydroxyanthranilate 3,4-dioxygenase (3-HAO). ACMS can be transformed either into picolinic acid (PIC) by an aminocarboxymuconate-semialdehyde-decarboxylase (ACMSD) catalyzed reaction, or it can form the NAD+ and NADP+ precursor quinolinic acid (QUIN) *via* a non-enzymatic conversion. QUIN is a key figure in excitotoxicity mediated neurodegeneration (52, 57, 58).

In light of the numerous enzymes participating and metabolites generated, the involvement of the KP in various disorders is not surprising. Indeed, changes in KP enzyme activity and metabolite levels have been detected in inflammatory, autoimmune, neurodegenerative and psychiatric diseases, as well.

In the following sections of this review we will briefly consider observations that point to existing links between KP and immune function. Then we will overview results obtained by models in which the KP was modulated by interventions effecting gene activity. Finally, we list known genetic alterations in genes of KP enzymes that are believed to be associated with changes in immune functions.

## Observations Indicating Links Between the Kynurenine Pathway and Immune Functions

### Indoleamine 2,3-Dioxygenase

Interplay between several enzymes of the KP and immune function are well-demonstrated. In this respect IDO, a key enzyme of the pathway, deserves particular attention. IDO is believed to exert its effects on immune function both by direct and indirect mechanisms. As an enzyme, IDO plays a role in Trp utilization and through this, in cellular metabolism *via* mTOR and GCN2 linked pathways. By converting Trp to KYN, IDO has a central role in determining concentrations of KP metabolites, many of which are direct or indirect regulators of immunofunction. Furthermore, IDO also acts as a signal protein. In concert with TGFβ, it regulates activation through non-canonical NF-κB response elements, thus affecting of its own production as well (45, 46).

**Figure 2:**
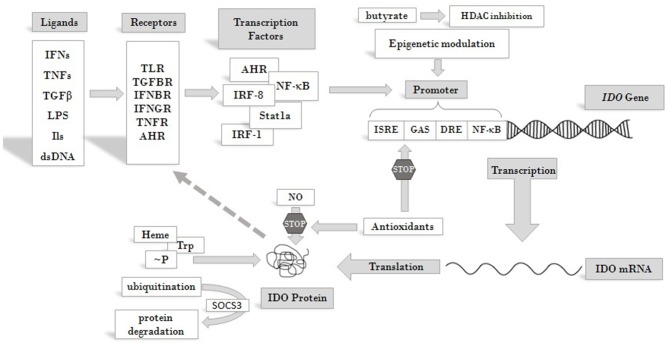
Overview of pathways leading to IDO enzyme production and regulation. IDO activity is regulated at different levels. Its substrate Trp and cofactor heme are positive regulators of the enzyme, whereas antioxidants and NO act as inhibitors. Phosphorylation at tyrosine side chains and ubiquitination modulate IDO activity and half life. At the level of transcription several cis regulatory elements of the IDO promoter collect regulatory signals via binding of transcription factors and epigenetic regulators, which respond to signals arriving from receptors that are activated by cytokines and other immunomodulatory molecules. Extracellular signals produced by other cells or pathogens and intracellular signals, such as cytosolic dsDNA can both induce IDO expression and feed-back regulation of the production has also been described [see text for details and (60)]. Abbreviations: IFN: interferon; TNF: tumor necrosis factor; TGFβ: transforming growth factor beta; LPS: lipopolysaccharide; dsDNA: double strand DNA; TLR: toll-like receptor; TGFBR: transforming growth factor beta receptor; IFNBR: interferon beta receptor; IFNGR: interferon gamma receptor; TNFR: tumor necrosis factor receptor; AHR: aryl hydrocarbon receptor; ISRE: IFN stimulated response element; DRE: dendritic cell response element; GAS: gamma-activated sequences; HDAC: histone deacetylase; ~P: phosphorylation; IDO: indoleamine 2,3-dioxygenase; SOCS3: suppressor of cytokine signaling 3; Trp: tryptophan; NO: nitrogen oxide.

IDO production and activity is controlled at different levels, including both transcriptional and post-translational regulation [reviewed in (46, 59)] (Figure 2). At the protein level, both its substrate, Trp, and its co-factor, heme, enhance IDO activity (61, 62). NO was found to reversibly inhibit the enzyme by binding to the active site (63, 64). Antioxidants also inhibit enzyme activity, both at transcriptional and post-transcriptional levels (62). Phosphorylation of two tyrosine side chains also can modulate IDO activity and its halflife (65). Decrease in IDO enzyme levels can be the result of ubiquitylation of the protein by the suppressor of cytokine signaling 3 (SOCS3) factor and proteosomal degradation (66).

At the level of transcription several cis-regulatory elements in the *IDO* promoter transmit regulatory signals. These are IFN stimulated response elements (ISRE), palindromic gamma-activated sequences (GAS), dendritic cell response elements (DRE) and non-canonical NF-κB binding sites [see reviews (60, 65)]. A number of transcription factors have been identified so far, which bind to these elements and play roles in the transcriptional regulation of IDO. Among them are IRF-1, IRF-8, Stat1a, NF-κB (67) and aryl hydrocarbon receptor (AHR). Recently, epigenetic regulation of the gene through histone deacetylase activity has also been reported (68). Through these factors various receptor-ligand pathways converge to determine *IDO* gene expression. These transmit regulatory signals from activated toll-like receptors (TLRs), transforming growth factor beta receptors (TGFBRs), AHR, interferon beta and gamma receptors (IFNBR and IFNGR), and members of the tumor necrosis factor receptor superfamily (TNFRs). Activation of any of these receptors by their ligands can trigger signaling pathways that promote or maintain the expression of *IDO*. Consequently, inflammatory signals, such as IFNs, lipopolysaccharides (LPS), interleukins (ILs) (such as IL-1, IL-2, IL-27, IL-10) TNFs, TGFs, and prostaglandins, can induce IDO production (69, 70). Thus, induction of the enzyme can be very complex and cell type specific [reviewed in (71)]. Moreover, some inflammatory markers act synergistically to increase IDO production and the types of cytokines affecting gene expression may differ in various cell types. This might be reflected by seemingly contradictory reports on the roles of particular ligands in IDO induction. According to some data, IFNγ is one of the main inducers of IDO expression (72). On the other hand, results obtained in LPS induced systemic inflammatory rat model did not support the role of IFNγ in IDO induction in the CNS and a more important role for other inflammatory cytokines, such as TNFα and IL-6, was proposed. Strengthening this conclusion, in LPS-stimulated glial cell cultures an increase of IDO expression was observed, accompanied by elevated levels of TNFα and IL-6, but no IFNγ expression. Based on these observations, it was concluded that IDO induction in the CNS by LPS is not mediated by IFNγ (73). However, recent findings strongly argue for the role of IFNs in the activation of IDO expression. It was found that not IFNγ but IFNα signaling was essential in enhancing IDO expression after B7 ligation of CTL4-Ig (74). *IDO* expression up-regulation *via* CpG oligodeoxynucleotide binding to TLR9 was also IFNα dependent (75). Futhermore, *IDO* expression was found to be upregulated by cytosolic DNA *via* the STING/IFNαβ pathway (76). Thus, it seems firmly established that type I IFNs play a cardinal role in enhanced *IDO* gene expression with inflammatory signals and that *IDO* expression following LPS treatment is induced by type I IFNs.

**Figure 3:**
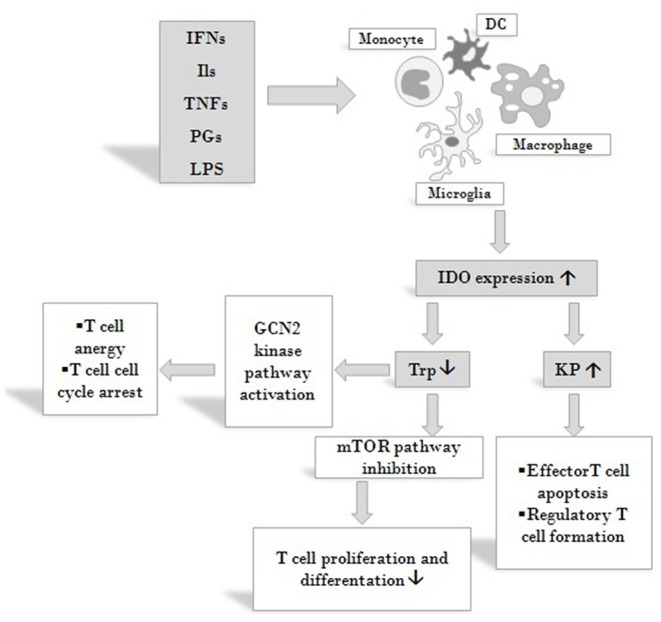
Effects on IDO activity on immune responses. IDO is expressed by various cells of the immune system in response to activation by inflammatory markers such as IFNs, ILs, TNFs, PGs and LPS. By decreasing the amount of available Trp IDO activates the GCN2 stress-kinase pathway leading to T cell anergy and cell cycle arrest, inhibits the mTOR pathway thus diminishing T cell proliferation. By increasing KP metabolite concentrations IDO also contributes to the apoptosis of effector T cells and promotes the formation of T cells of the regulatory subtype. Via these mechanisms IDO might exert profound effects on both systemic and local immune responses. Abbreviations: IFNs: interferons; Ils: interleukins; TNFs: tumor necrosis factors; PGs: prostaglandines; LPS: lipopolysaccharide; DC: dendritic cell; IDO: indoleamine 2,3-dioxygenase; Trp: tryptophan; KP: kynurenine pathway; mTOR: mammalian target of rapamycin.

IDO is expressed by numerous cells of the immune system: monocytes, dendritic cells (DCs), macrophages and microglia (48). It regulates immune responses in various direct and indirect ways (Figure 3). On the one hand, by decreasing the amount of available Trp, it causes an increase in free transfer RNA, thus activating the GCN2 stress-kinase pathway leading to T cell anergy and cell cycle arrest (77). On the other hand, a lack of the amino acid leads to the inhibition of the rapamycin (mTOR) pathway followed by a translational block (78). Moreover, *via* the formation of different immunologically active kynurenine metabolites, IDO also contributes to the apoptosis of effector T cells and promotes the formation of regulatory T cells (59, 79).

Another important link between KP and the immune system is manifested by DCs, in particular in their role in inflammatory processes. Sepsis is a systemic inflammatory response syndrome which leads to hemodynamic shock accompanied by multi-organ failure. It is a major cause of mortality and morbidity among hospitalized patients. Sepsis is the consequence of microbial infection, in which Gram-negative bacteria outer-membrane components (LPS) trigger the uncontrolled production of pro-inflammatory cytokines, which leads to the imbalance of pro-and anti-inflammatory factors. DCs seem to play a cardinal role is sepsis development, as they are capable of producing pro- (IL-12) and anti-inflammatory (IL-10) cytokines, the balance of which was found to be altered during infection (80). In DCs, IDO expression is induced by LPS and the enzyme production contributes to the imbalance of anti- and pro-inflammatory cytokines (2).

A growing body of data shows the involvement of IDO in immune responses to tumors [see in (45, 46, 81)]. A pivotal role of the enzyme is seen in establishing the immunosuppressive microenvironment of tumors by altering the functions of infiltrating T lymphocytes, thus promoting immune escape and progression of cancer cells (82, 83). Upregulated expression of IDO has been reported in the microenvironment of laryngeal and esophageal carcinomas (84–87) and higher plasma enzyme activity was reported in lung-, gynecological-, breast- and colorectal cancers, and melanoma. Both local expression changes and elevated plasma IDO activity was reported in patients with nasopharyngeal carcinoma (NPC) (81). Interestingly, a significant difference in plasma IDO levels could be detected between healthy controls and NPC patients with metastasis, in contrast to patients without metastasis. Plasma IDO activity was also found to have a prognostic value, as patients with higher levels of enzyme activity had significantly lower rates of survival compared to those with lower IDO activity. Higher enzyme activity was shown to result from higher expression levels: Fukuno et al. reported that IDO mRNA expression in patients with acute myeloid leukemia (AML) was associated with a worse disease outcome (88). In light of the role IDO plays in immune responses to cancer, it is no wonder that IDO modulation is a hot topic in cancer research. Many therapeutic approaches are underway for pharmaceutical enzyme inhibition (65). This review will not discuss these in detail since our aim is to give an overview of findings on approaches targeting the KP by gene modulation.

### Tryptophan-Dioxygenase

The first step of the KP can also be catalyzed by TDO, a functional ortholog enzyme of IDO. However, while IDO is mainly expressed by various immune cells, thus regulating the amount of locally available Trp, TDO is expressed in the liver, affecting the systemic level of the amino acid. The activity of the enzyme can be regulated by various mechanisms. *TDO* transcription is enhanced by glucocorticoids and this is potentiated by glucagon, but inhibited by adrenaline and insulin (89). TDO can also be activated by its cofactor, heme, and its substrate, Trp [reviewed in (61)]. Recent evidence demonstrates TDO presence in rat skin and the CNS of humans (90, 91), thus broadening the location and raising further questions on the exact role of the enzyme. As Trp stabilizes the TDO enzyme complex (92), and cortisone, a hormone with anti-inflammatory effects, enhances TDO expression (93, 94), one can expect the involvement of the enzyme in immune processes. This was reported first in the early 2000s (95, 96): in 2000, Tatsumi et al. proposed a role for the enzyme in tolerance during embryonic implantation, based on finding upregulated expression of TDO mRNA in murine decidualized stromal cells surrounding the implanted embryo (96). In 2001 Suzuki et al. reported high TDO expression during early murine gestation, preceding the expression of IDO, thus revealing an important role of TDO in fetal tolerance (95). When regarding the immune modulator effects of the KP, research has been mainly focused on IDO, but a growing body of evidence is accumulating on the involvement of TDO as well. It indicates the presence of the enzyme in tumor immune resistance (38, 97) and parasite, viral and microbial infections (98) (Table 5). Expression of TDO by several different tumor types—such as melanomas, bladder-, and hepatocarcinomas—drew attention to the possible role of the enzyme in tumor immunity. TDO was found to be constitutively expressed in glioblastomas and excessive production of the AHR agonist KYN was found to contribute to the immune escape, higher motility and survival of tumor cells (38).

### Kynurenine Monooxygenase

A third enzyme with assumed immunomodulatory effects of the KP is KMO, which is situated at an important branch point of the pathway. KYN can be catalyzed by KATs into KYNA, representing a neuroprotective and antioxidant branch of the pathway. On the other hand, KMO can convert KYN to 3-HK, which can be further converted into PIC and QUIN. Both of these metabolites are known to have neurotoxic and free radical generating properties. Thus, KMO has a key role in determining the balance between pro- (QUIN, 3-HK, PIC) and anti-inflammatory (KYNA) kynurenine metabolites.

The substrate of the KMO enzyme, KYN, was shown to promote tumor formation and the generation of regulatory T cells *via* AHR (99) and adenylate- and guanylate-cyclase pathway activation (38). However, the mode of action of KYN on AHR raised questions, as the structure of the metabolite does not show the necessary features for high-affinity AHR binding. Recently two KYN condensation products have been identified, which are high affinity AHR ligands, active at low picomolar levels. Thus, KYN seems to be a pro-ligand that spontaneously converts to derivatives possessing AHR agonist properties (100). Theoretically, enhancing the metabolism of KYN *via* KMO upregulation could be protective against the development of tumors. However, upregulation of KMO also leads to the formation of metabolites with reactive oxygen species (ROS) generating properties, such as 3-HK, 3-HAA, and QUIN. QUIN also exerts excitotoxic effects through the activation of NMDARs (38). Pharmacological inhibition of KMO enhances the production of KYNA, a metabolite with neuroprotective effects. Besides its neuroprotective effects (101, 102) KYNA also has an important role in immunomodulation, mainly *via* the activation of GPR35 receptors and AHRs [reviewed: (103)]. KYNA was found to attenuate inflammation under inflammatory conditions by several means: by reducing TNF expression in monocytes, IL-4 secretion of T-cell receptor stimulated variant natural killer-like T cells and LPS induced IL-23 formation of DCs [reviewed: (103)]. The expression of KMO was also found to be upregulated in the CNS of rats in LPS induced systemic inflammation, together with a significant increase in pro-inflammatory cytokines such as TNFα and IL-6 (73).

KMO expression and activity have been investigated in autoimmunity related diseases. A link seems to exist through AHRs as these receptors play an important role in the regulation of pro-inflammatory Th17 cell differentiation (42) and Trp metabolites act as agonists of AHRs. KP metabolites play roles in promoting the differentiation of naive T cells into effector Th17 cells (104), which are governors of autoimmune diseases (105). Stephens et al. reported that Th17 cells highly express KMO, and that either the inhibition of the enzyme or the addition of 3-HK augmented the formation of effector T cells (42).

Since these three enzymes of the KP seem to be associated with immune functions, genetic approaches aimed at modifying immune responses are focused on altering their expression. So far, primarily gene knockouts, gene expression regulation by small interfering RNA (siRNA), short hairpin RNA (shRNA) and different gene delivery techniques into model animals and tissue culture cells have yielded results regarding the immunomodulatory effects of these enzymes. The next section will review these results. We find it important to point out here that with the rapid progress of gene modulatory and gene editing techniques it is expected that the data summarized here will grow in the near future.

## Immunomodulation *via* Altering the Expression of Genes That Code for Enzymes of the Kynurenine Pathway

### Indoleamine 2,3-Dioxygenase

#### Effects of Genetic Modification of Indoleamine 2,3-Dioxygenase Related to Immune Responses in Infection

During the course of sepsis, induction of IDO by bacterial endotoxins plays a pivotal role in the disproportional production of pro- and anti-inflammatory cytokines. Consequently, the detrimental effects of excessive pro-inflammatory stimuli could be lessened by the genetic inhibition of IDO (2). Indeed, in IDO knockout mice (IDO^−/−^) the balance was shifted toward the production of the anti-inflammatory IL-10. IDO inhibition had beneficial effects on the survival rates as well: overall survival from LPS induced shock was higher among IDO^−/−^ animals compared to wild-type mice (2) (Table 1).

In mouse model, Blumenthal et al. found upregulation of *IDO* expression upon *Mycobacterium tuberculosis* infection both *in vitro* and *in vivo*. Though *in vitro* experiments indicated that genetic ablation of the enzyme resulted in enhanced T cell proliferation after infection, such changes were not observed *in vivo*, as there was no significant difference between the numbers of activated T cells in the lungs and lymph nodes of IDO^−/−^ and IDO^+/+^ animals. In accordance with this, the survival rate of *Mycobacterium tuberculosis* infected IDO^−/−^ mice did not differ significantly from that of their IDO expressing counterparts (8). Uropathogenic *Escherichia coli* (UPEC) was also found to elevate IDO expression *in vitro* in human uroepithelial cells and polymorphonuclear leukocytes. In mice *in vivo* genetic ablation of the enzyme (IDO^−/−^) resulted in increased levels of pro-inflammatory cytokines, such as granulocyte-colony stimulating factor, IL-6 and IL-17, leading to an increase in granulocyte accumulation and local inflammation in the bladder of animals and decreased survival of extracellular bacteria as compared to wild type (IDO^+/+^) mice. These observations led to the conclusion that *via IDO* up-regulation the pathogen reduces host inflammatory responses thus enabling its own survival (9). Similarly to UPEC, infection with *Rhodococcus equi*, a facultative intracellular pathogenic bacterium also enhanced *IDO* expression in DCs and alveolar macrophages. In the liver tissue of *IDO*^−/−^ animals, infection with the pathogen decreased TGFβ level and FOXP3 expression, indicating a reduction in T regulatory cell responses, in parallel with prolonged liver inflammation (10).

The IDO immunomodulatory role was also studied in chronic viral diseases. Infection of mice by LP-BM5 Murine Leukemia Virus (MuLV) resulted in the development of fatal immunodeficiency syndrome, also known as murine AIDS. Similarly to acquired immunodeficiency in humans, murine AIDS is characterized by activation and proliferation of T and B cells with altered functions, a decrease in the number of function of natural killer (NK) cells and abnormal cytokine production. Animals suffering from the disease are more prone to developing B cell lymphoma and to opportunistic infections. Genetic inhibition of IDO was found to evoke protective effects in MuLV infected animals: in IDO^−/−^ mice an increase was observed in the levels of type I IFNs and the number of plasmocytoid dendritic cells (premature type of DCs, pDCs) accompanied by a decrease in virus replication as compared to wild type animals. Interestingly, type I IFN neutralization in IDO deficient animals abolished the decrease in virus replication, suggesting a cardinal role of these IFNs in immune responses against viruses (3). The enhanced production of type I IFNs was attributed to pDCs, which were earlier reported to produce a number of different type I IFNs upon viral infection (106), and are chronically up regulated in HIV patients too (107). According to a further report from the same laboratory, genetic inhibition of IDO expression was also beneficial with the encephalomyocarditis virus (ECMV) murine model of acute viral myocarditis (4). Similarly to the case of MuLV infected mice, IDO^−/−^ animals showed a significantly higher survival rate after ECMV infection compared to WT mice. In knockout animals, ECMV replication was inhibited, as demonstrated by the lower levels of viral genomic RNA in the heart and consequently the decreased levels of myocardial damage. The mechanism of the protective effect in the absence of the enzyme is believed to be the lack of KYN and 3-HK production. These metabolites are proposed to decrease the production of antiviral type I IFNs, key factors in myocardial damage protection. Indeed, treatment of IDO^−/−^animals with these KP metabolites decreased the otherwise elevated levels of type I IFNs and led to increased myocardial destruction and a prominent reduction in the survival rate. Based on the findings that bone marrow transplantation from IDO^−/−^ animals to IDO^+/+^ mice resulted in significantly higher IFNβ levels than was in the case with IDO^−/−^ animals receiving bone marrow from WT animals, it was proposed that type I IFN production is regulated in the bone marrow. It was concluded that inhibition of antiviral type I IFN production by IDO is the result of multiple mechanisms: on the one hand, the number of activated macrophages is suppressed by the formation of KP metabolites, and on the other hand, local Trp depletion also can contribute to a decrease in type I IFN production (4). In pDCs type I IFN production is regulated by the mTOR pathway (108) that can be antagonized by amino acid starvation (109). Considering that IDO catalyses the metabolism of Trp, one can suppose that the enzyme inhibits mTOR signaling *via* locally depleting Trp (4).

IDO overexpression was also found to be beneficial in the course of West Nile virus infections. Using IDO expressing HeLa cells, it was shown that overexpressing IDO prior to viral infection resulted in a significant decrease in viral replication. Excessive enzyme expression restricted the spread of the infection to the neighboring cells (6).

In contrast with the findings of Hoshi and colleagues, O'Connor et al. reported that IDO deficient LP-BM5 infected mice displayed similar disease severity to their IDO expressing counterparts. These authors detected no differences in retroviral load between IDO^−/−^ and WT animals, thus the lack of enzyme did not seem to affect viral replication or viral spread (7). Similar results were reported by Huang et al., as they found no significant effect of either pharmacological or genetic inhibition of IDO on the outcome and severity of MuLV infection. On the other hand, this study identified IDO as a major factor in pain hypersensitivity related to acute influenza A and MuLV infection. IDO was found to enhance hypersensitivity *via* the production of KYN, and genetic inhibition of IDO resulted in the alleviation of acute and chronic pain related to infection (5).

#### Effects of Genetic Modification of Indoleamine 2,3-Dioxygenase on Immune Responses in Autoimmune and Allergic Diseases

Besides inflammation related to bacterial and viral infections, the role of IDO in autoimmune and allergic processes has also gained attention (Table 2). Studies on the role of IDO in mucosal allergic processes revealed that though the enzyme is not essential for antigen-induced airway immune tolerance, it plays a cardinal role in antigen-induced Th2 mediated immune responses (11). Genetic inhibition of the enzyme in a mouse model of acute allergic airway inflammation led to a decrease in effector T cell formation and in the production of Th2 cytokines, such as IL-4, IL-5, IL-9, and IL-13, which play important roles in asthma and other allergic diseases (110). As a consequence, attenuation in airway inflammation, mucus secretion, airway eosinophilia and hyperresponsiveness was observed in IDO^−/−^ animals when compared to their WT counterparts. Investigation of a chronic asthma model yielded similar results with fewer DCs in the lymph nodes and a decrease in parameters indicating allergic airway inflammation. In accordance with this, IFNγ (a Th1 type cytokine) expression was elevated. In summary, IDO expression of infiltrating DCs seems to be essential in promoting Th2 type immune response upon exposure to airway allergens (11).

In contrast with the above, no effect of knocking out IDO was found in an auto-inflammatory disease, systemic juvenile idiopathic arthritis (sJIA) (12). In a number of sJIA cases secondary hemophagocytic-lymphohistiocytosis (sHLH) develops, a condition characterized by the over-activation of macrophages [macrophage activation syndrome (MAS)], thus leading to a potentially fatal disease state. The development of a cytokine storm—in which IL-1β, IL-6, and IL-18 play cardinal roles—is characteristic of both sJIA and sHLH (111). It has been supposed that both sJIA and sHLH were the consequences of the lack of effective down regulation of an exaggerated immune response (112, 113). Taking into account the immunomodulatory effects of IDO, assuming the involvement of the enzyme in these disease states seemed well-grounded. However, results of Put et al. did not support this hypothesis. Genetic inhibition of IDO in mice models of sJIA, MAS, and sHLH, did not indicate differences in the symptoms of IDO^−/−^ animals as compared to WT mice. Though neither the level of IDO2 nor TDO was found to be elevated, they hypothesized that the absence of IDO was compensated by other Trp metabolizing enzymes of the KP (12).

According to results reported by Lemos et al., the presence of *IDO* is essential in provoking beneficial immune responses in a mouse model of experimental autoimmune encephalitis (EAE) (13). Cytosolic DNA leads to the activation of the Stimulator of Interferon Genes (STING) adaptor and results in the induction of interferon type I (IFNαβ) production. Continuous activation of STING provokes autoimmunity due to a failure in immune tolerance. In an EAE mouse model of multiple sclerosis (MS), it was found that following immunization with myelin oligodendrocyte glycoprotein (MOG), systemic treatment with DNA nanoparticles (DNPs) or cyclic diguanylate monophosphate (c-diGMP) induced STING signaling. In this way, potent regulatory immune responses could be achieved leading to restrained EAE severity and delayed disease onset. In accordance with this, reduced levels of effector T cell infiltration in the CNS and decreased immune responses to the administered MOG therapy in the spleen were observed. Interestingly, MOG treatment stimulated CNS neurons to express IDO, however, after DNP therapy no IDO expression could be detected in the CNS of immunized mice. The authors concluded that while immunization with MOG led to IDO expression in neurons, DNP induced the enzyme in tissues outside the CNS and paradoxically diminished MOG induced IDO expression in neurons. Based on these findings it was proposed that IDO induction in lymphoid tissues inhibited infiltration of effector immune cells in the CNS and consecutive neuronal IDO expression. In accordance with the above, for therapeutic responses *IDO* gene function was essential only in hematopoietic cells and lack of *IDO* in non-hematopoietic cells did not cause changes in the outcome of DNP therapy. The changes provoked by DNP administration were found to be highly dependent on intact *IDO* and IFNαβ receptor genes, as no therapeutic responses were observed in either STING-KO or IDO^−/−^ animals. It was concluded, that attenuation of immune responses upon DNPs and c-diGMP application was due to the induction of T cell regulatory responses *via* the STING-IFNαβ-IDO pathway. This, accompanied by elevated Trp degradation and changes in the balance of pro-and anti-inflammatory metabolites and cytokines, results in better immune response outcome (13). Earlier reports on diminished Treg responses, exacerbated EAE disease severity and increased encephalitogenic Th1 and Th17 responses in IDO^−/−^ mice supports this notion. Administration of the Trp metabolite 3-HAA, besides inhibiting Th1 and Th17 cells, also enhanced Treg cell responses, thus improving disease outcome. It was concluded that IDO, by promoting the formation of Trp metabolites, such as 3-HAA, enhances Treg differentiation (14).

Investigation of IDO mRNA expression in mice with collagen induced arthritis (CIA), an animal model of rheumatoid arthritis (RA), revealed a significant increase in the level of the transcript in the lymph nodes of affected animals. Enhanced IDO expression was mainly limited to DCs in lymph nodes. By comparing disease progression in IDO deficient to WT mice, it was found that though the severity of the disease was similar at the early stages, in WT animals a plateau was observed 5 days after disease onset, while in IDO^−/−^ mice arthritis progressed further leading to a more severe disease. Increased joint damage, higher production of IFNγ and IL-17 in the lymph nodes and higher Th1 and Th17 cell frequency were observed in paws of IDO deficient animals. These observations led to the conclusion that IDO activation in lymph nodes is essential in reducing the accumulation of Th1 and Th17 cells in joints and thus restraining disease severity and progression in RA animal model (15).

In accordance with the findings of Criado et al., Chen et al. reported that adenoviral vector-mediated intra-articular IDO gene delivery (AdIDO) into ankles of CIA rats ameliorated disease severity. In the ankle joints of CIA animals, a significant reduction was observed in bone destruction, soft tissue swelling and synovial hyperplasia. Furthermore, a significant decrease in CD4+ T cell infiltration accompanied by a higher apoptosis rate and reduced CD68 macrophage infiltration was detected in AdIDO treated CIA animals. Reduced Th17 cell activity was found as well, as was indicated by diminished IL-17, IL-6, IL-1β concentrations and RORγt expression in ankle joints and draining lymph nodes. The authors concluded that *IDO* gene therapy reduced arthritis *via* the up-regulation of the Trp degradation pathway, thus increasing kynurenine concentrations, leading to increased CD4+ T cell apoptosis and diminished IL-17 production (16).

Type I diabetes is an autoimmune diseases, in which insulin-producing pancreatic cells are destroyed by activated T lymphoctyes (114). In pDCs IDO expression is triggered by TGFβ *via* the non-canonical NF-κB pathway. Besides its Trp catabolizing ability, in pDCs IDO acts as a signaling molecule as well: promoting its own and also TGFβ expression, it amplifies immune tolerance and enables the spreading of TGFβ dependent tolerance (115–117).

In a study of non-obese diabetic (NOD) mice, the animal model of autoimmune diabetes, TGFβ failed to activate the non-canonical NF-κB pathway, thus no up-regulation could be achieved in *Ido* expression. However, after transfection of the *Ido* gene into NOD pDCs, TGFβ administration led to the activation of the NF-κB pathway. This enhanced IDO expression was accompanied by a decrease in IL-6 and TNFα pro-inflammatory cytokine production and an up-regulation of the anti-inflammatory TGFβ, ensuring a more immune-tolerant setting. Enhanced IDO expression also led to decreased production of pancreatic β-cell auto-antigens. It was concluded, that immunoregulatory functions of TGFβ require a basal expression level of IDO, which could be achieved by the forced expression of the enzyme (17). The observation that enhancement of both the enzymatic and signaling activity of IDO proved to be beneficial in NOD mouse model, might allow us to expect success from IDO modulation in other, autoimmune diabetes related disorder as well.

A study reported by Ravishankar et al. further strengthens the role of IDO in the course of autoimmune diseases. In a model of Lupus-prone Murphy Roths large (MRL*lpr/lpr*) mice—an analog of systemic lupus erythematosus (SLE)—significant constitutive IDO expression was observed in the spleen of pre-symptomatic MRL*lpr/lpr* animals. In contrast, in normal mice little basal IDO activity was present. Treatment of MRL*lpr/lpr* mice with pharmacological IDO inhibitor, D1MT, yielded significantly elevated autoantibody levels and IgG immune-complex deposition in the skin and kidneys of affected animals, what is a manifestation of loss of self-tolerance. Injecting apoptotic thymocytes in IDO^−/−^ MRL*lpr/lpr* animals resulted in an increase in autoantibody titers, pro-inflammatory cytokine production, and dysregulated T cell responses culminating in lethal autoimmunity. On the other hand, exposure of IDO^+/+^ MRL*lpr/lpr* mice to apoptotic cells did not lead to pathogenic autoimmunity, as the response to thymocytes was low and self-limiting. Whether the presence of IDO enables the suppression of T cell responses to the antigens presented or whether it inhibits the antigen presentation itself to potentially autoreactive T cells, needs further elucidation. Nevertheless, the role of IDO in the maintenance of immune homeostasis and in the prevention of autoimmune progression is inevitable (18).

#### Effects of Genetic Modification of Indoleamine 2,3-Dioxygenase on Transplant Related Immune Responses

Besides studies on the role of IDO in immune responses to infections and autoimmune reactions, its involvement in transplant responses is also a focus of research (Table 3). A possible way of treating autoimmune (Type I) diabetes could be the restoration of insulin production *via* the transplantation of insulin producing pancreas cells (118). However, major concerns are the reappearance of autoimmunity and the rejection of the allograft (119). A study by Alexander et al. yielded promising results with respect of these issues. They found that transplantation of diabetic mice with pancreatic islets expressing IDO *via* adenoviral gene transfer resulted in prolonged graft survival (19). *In vitro* experiments revealed a significant depletion of locally available Trp and inhibition of the proliferation of T cells obtained from diabetic animals. The extended *in vivo* graft survival was proposed to be due to local Trp depletion at the site of transplantation, in accordance with the *in vitro* findings. These results suggest that transplanted pancreatic cells expressing IDO due to *ex vivo* genetic editing are capable of inhibiting the proliferation of host diabetic T cells, thus preventing graft rejection (19). These findings open new possible avenues in the treatment of type I diabetes.

Enhanced IDO expression was also found to be beneficial in the case of transplantation of an immune-privileged tissue, the cornea (20). Over expression of IDO in donor corneal endothelial cells prior to the transplant resulted in increased formation of L-KYN in the allograft. As a consequence, the proliferation of allogeneic T cells was locally inhibited, thus permitting the prolonged survival of the graft when compared to no IDO expresser controls. Similarly, enhanced IDO expression prevented lung allograft injury in a rat model. Liu et al. used non-viral gene transfer methods to deliver human IDO gene to enhance IDO expression in the transplanted lungs. They found that both functional and histological properties of IDO overexpressing lungs were significantly improved in comparison to allografts without enhanced gene expression (21, 23). IDO gene delivery blocked local T cell responses, but could not prevent the recruitment of neutrophil granulocytes. Enhanced IDO expression led to the inhibition of intracellular ROS formation, thus reducing ROS induced necrosis and apoptosis of lung cells (21). IDO overexpressing lung allografts displayed more preserved bronchus-alveolar architecture due to significantly less interstitial and peribronchial collagen deposition than controls. *In vitro* IDO expressing lung cells inhibited the TGFβ mediated proliferation of fibroblasts, however, this could also be prevented by the addition of Trp, suggesting that local Trp depletion due to enhanced IDO expression was the mechanism of fibroblast proliferation prevention (23).

In following studies, Liu et al. found that in transplanted lung allografts IDO overexpression reduced the number of infiltrating CD3+ and CD8+ T cells. CD8+ T cells lost their cytotoxic properties, and a significant reduction was observed in their TNFα and IL-2 production. *In vivo* findings revealed that IDO overexpression limited ATP production in CD8+ cells. *In vitro* studies showed that IDO selectively diminished the activity of electron transport chain complex I, which explains the reduced ATP production of infiltrating T cells (22).

Further studies revealed that besides enhanced IDO expression, systemic administration of a KP metabolite, 3-HAA was also capable of prolonging lung allograft survival. Furthermore, IDO overexpression in lung allografts, in addition to TNFα and IL-2, also decreased the level of IFNγ, IL-12, IL-4, IL-5, IL-6, and IL-13. However, there was no reduction in the level of a potential protective cytokine, IL-10. As IL-2, IL-4, IL-12, and IL-6 play important roles in the production of effector memory T cells, it was proposed that IDO overexpression inhibited not only early T cell responses, but also diminished the formation of memory T cells, thus prolonging the survival of the allograft. *In vitro* findings demonstrated that high IDO environment led to decreases in intracellular calcium levels, phospholipase C-γ1 phosphorylation and mitochondrial mass. These observations offer novel insight into the mechanisms by which IDO exerts T-cell inhibiting properties: namely by impairing T cell receptor activation *via* decreasing calcium influx, thus impairing calcium signaling (26).

IDO overexpression in fibroblasts diminished CD3+ T cell recruitment at cutaneous wounds as well. The *in vitro* model showed that wounds receiving IDO expressing human fibroblasts had faster healing rates compared to those grafted with non-treated fibroblasts. This was partly because of the significantly increased vascularisation in wounds prior to receiving IDO expressing fibroblasts as observed in an *in vivo* rat model. However, the addition of Trp diminished otherwise enhanced angiogenesis, implicating the Trp depleting role of IDO in the course of capillary formation (24).

Overexpression of the enzyme was found to be beneficial regarding cardiac allograft survival as well. Overexpression of IDO in DCs resulted in decreased allogeneic T cell proliferation *in vitro*. Based on *in vivo* experiments it was concluded that adenovirus mediated IDO gene transfer in the donor heart led to decreased monocytes, macrophages and T cells infiltrating the organ. This was accompanied by diminished intragraft IFNγ, TNFα, TGFβ, and IL-1β levels and prolonged graft survival (25).

Altogether, these findings underline the feasibility of using IDO gene induction for the purpose of preventing allograft rejection.

#### Effects of Genetic Modification of Indoleamine 2,3-Dioxygenase on Immune Responses in Disease States Related to Chronic Inflammation and in Intestinal Immunity

In addition to its possible use to modulate immune processes related to infectious diseases, allergy, transplantation, and autoimmunity, the involvement of IDO has also been investigated in diseases accompanied by chronic inflammation, such as diabetes, aorta aneurysm, obesity, and hepatic fibrosis (Table 4).

Hyperglycaemia induced chronic retinal inflammation has a pivotal role in the development of diabetic retinopathy (DR), one of the major causes of visual impairment worldwide (120). In a recent study, Nahomi et al. reported a 50 percent increase of the level of IFNγ in human diabetic retinas accompanied with elevated IDO expression (27). Genetic inhibition of IDO function in diabetic IDO^−/−^ mice was found to reduce retinal capillary degeneration, as acellular capillary formation in knockout mice was alleviated as compared to their WT counterparts (27).

Chronic inflammation has been reported to be a primary feature of atherosclerosis as well (121). The higher angiotensin II (AngII) plasma levels in atherosclerosis suggest hormone involvement in the development of various cardiovascular diseases. This raised the possibility of using the hormone to generate animal models of diseases linked to atherosclerosis. Indeed, infusion of Apolipoprotein E knockout (ApoE^−/−^) mice with AngII led to the development of more severe atherosclerotic lesions in the aorta. In the affected aortic segments, high numbers of lipid-laden macrophages and lymphocytes were observed accompanied by increased macrophage infiltration in the adventitia (122). In a mouse model of atherosclerosis, AngII was found to enhance the expression of IDO in parallel with increased IFNγ expression, indicating a link between the KP and arterial degeneration (28). Inhibition of the enzyme exerted beneficial effects, as in WT mice AngII infusion resulted in increased oxidative stress, dysfunction, and apoptosis of endothelial cells, however, these detrimental effects were all suppressed in IDO^−/−^ animals. AngII infusion also increased plasma kynurenine levels in WT animals, however, such changes were not observed in IDO deficient ones. *In vitro* studies revealed that upon IFNγ induced Ido activation, 3-HK is formed, which, by increasing nicotinamide adenine dinucleotide phosphate (NAD(P)H) oxidase activity, leads to enhanced ROS production, triggering dysfunction and apoptosis of endothelial cells (28). These results propose a possible therapeutic approach to atherosclerosis linked cardiovascular diseases: genetic inhibition of IDO leading to reduced 3-HK and, consequently, diminished ROS production could be a feasible way of avoiding endothelial cell loss.

Abdominal aortic aneurysm (AAA) is a potentially fatal condition characterized by the abnormal dilatation of the abdominal aorta. The pathomechanism leading to the disease is similar to that seen in atherosclerosis, as it includes the apoptosis of vascular smooth muscle cells (VSMCs), degeneration of the extracellular matrix by a metalloproteinase mediated mechanism, collagen remodeling and chronic inflammation of the aortic wall (123, 124). In a hypercholesterolemic mouse model of AAA, in which low density lipoprotein—receptor deficient (Ldlr^−/−^) mice were infused with AngII and fed with high fat diet (HFD), the absence of IDO was found to be protective against the development of aneurysms (29). In IDO^−/−^ animals infused with AngII, TUNEL assay did not indicate increased levels of apoptosis, but α-actin staining was increased. Both observations suggest the protective effect of IDO exerted *via* the inhibition of apoptosis of VSMC. A comparison of circulating immune cells in IDO^+/+^ and IDO^−/−^ animals revealed no significant difference in the number of neutrophils, monocytes, CD4+ and CD8+ T cells or CD19+ B cells. Similarly, no significant difference was detected in infiltrating macrophages and T lymphocytes in the adventitia and media of the aortic aneurysm. However, IL-17 production was significantly decreased in IDO deficient animals as compared to their IDO expressing counterparts (29). In summary, these findings raise the possibility of a mechanism similar that seen in the development of atherosclerosis. As such, IDO mediated 3-HK formation could be one of the main culprits in arterial wall degeneration (28, 29).

Recently a further kynurenine metabolite and an enzyme of the pathway were identified to take part in the pathomechanism of the disease. According to a study by Wang et al., IDO knockout and siRNA mediated *Kynu* silencing in ApoE^−/−^ mice were protective against AgII induced AAA formation (30). In ApoE^−/−^ mice the genetic inhibition of both IDO and KYNU caused a decrease in elastic lamina degradation and aortic expansion was observed following AngII infusion. The comparison of serum inflammatory markers, such as IFNγ, TNFα, IL-6, and cyclophilin-A, revealed no significant differences between IDO expressing and IDO^−/−^ animals, suggesting another IDO regulated mechanism apart from immune mediation. 3-HAA was identified as a main factor in the pathomechanism of aneurysm development as it was found to upregulate the expression of matrix metallopeptidase 2 (MMP2), which has a central role in the pathophysiology of AAA formation *via* extracellular matrix degeneration (30, 125).

The production of 3-HAA was regulated by both IDO and KYNU. On the one hand, AngII infusion in IDO^+/+^ mice induced the expression of both enzymes, which resulted in the elevation of the level of 3-HAA both in the plasma and aorta of these animals, however, no such changes were observed in the absence of IDO. On the other hand, genetic inhibition of KYNU led to decreased 3-HAA production and diminished MMP2 expression, consequently preventing the formation of AAA (30). The investigation of human AAA samples revealed similar changes in the KP: both IDO and KYNU enzymes were significantly upregulated in human aneurysm samples, accompanied by higher levels of 3-HAA in the affected aortic wall (30). These findings underline the therapeutic potential of interfering in the pathway to prevent vascular degeneration.

Association between obesity, inflammation and the gut microbiome has been intensively investigated in the past decades (31, 126). In a recent study, Laurans et al. reported that IDO^−/−^ mice fed a high fat diet showed lower body weight and fat mass compared to WT animals on the same diet. Knockout animals also had lower liver weights accompanied by less lipid accumulation and decreased macrophage infiltration in the organ, implying the presence of a protective mechanism against steatosis. A decrease in inflammatory processes was also detected in white adipose tissue (WAT) of IDO^−/−^ mice compared to their wild type counterparts. In epididymal and inguinal adipose tissues, lower numbers of infiltrating macrophages were detected and in inguinal WAT the number of M2 type cells was higher, whereas there was no significant change in the number of M1 type cells (31). M2 macrophages are associated with alleviating inflammation, propagating wound healing and are regarded as a “benign” subtype in contrast with the pro-inflammatory, activated M1 type ones (127). In accordance with this, the levels of anti-inflammatory cytokines—such as IL-10, 4, and 5—were significantly higher in animals lacking the enzyme. Besides protection against liver steatosis, genetic inhibition of IDO also proved to be beneficial against the development of insulin resistance, as indicated by lower insulin concentrations measured during oral glucose tolerance tests (OGTT) and better results to insulin tolerance tests (ITT) by IDO^−/−^ animals compared to WT mice. These findings suggest the protective role of IDO inhibition against obesity and obesity related pathological changes in metabolism affecting the liver and glucose homeostasis. Laurans et al. also attempted to identify the causative role of IDO in obesity and related disorders. They found that KYN and KYNA supplementation did not abolish the positive effects of IDO deletion on body weight, thus it is unlikely that the beneficial metabolic changes seen in the case of IDO inhibition are the consequences of the lack of these metabolites (31). Several previous observations on obesity related intestinal dysbiosis and gut derived LPS translocation (128), the demonstration of high IDO expression in the gastrointestinal tract, and that activity of IDO was increased in the intestine of high fat diet animals (129) support the assumption that higher intestinal IDO expression leads to a shift toward kynurenine production instead of the formation of indole derivatives (31). In concert with this assumption, Laurans and colleagues found that in the IDO^−/−^ HFD fed mice higher intestinal levels of indole-3 acetic acid (IAA) were present. In parallel with this, the levels of two cytokines known to be dependent on indole derivatives (130), IL-17 and IL-22 [which both play primary roles in rapid immune response of the host against microbes (131)] were increased, accompanied by the decreased expression of inflammation related genes. These changes in the intestinal tract were accompanied by significantly diminished LPS levels in the plasma of IDO^−/−^ animals on HFD compared to WT HFD animals. All combined, these findings strongly suggest a causative effect of IDO deletion in maintaining an intact intestinal immune barrier in obesity (31).

However, while the absence of IDO can be beneficial, as in most of the cases cited above, the lack of the enzyme can also have detrimental effects in certain cases. The seemingly opposing findings demonstrate the diverse and complex role of the enzyme in the regulation of immune processes. Hepatic fibrosis is a consequence of chronic inflammation which can be triggered by various agents, such as viral infection, drugs, metabolic and autoimmune diseases (32). Elevated expression of IDO has been reported in hepatitis (132), leading to the assumption that the enzyme might be involved in hepatic fibrosis. Based on data of elevated levels of pro-inflammatory cytokines, such as TNFα and IL-6, in a hepatitis model, Ogiso et al. proposed that the induction of IDO by pro-inflammatory agents might play a role in the disease. In a carbon-tetrachloride (CCl4) induced animal model of the disease, the absence of IDO was found to aggravate the progress of fibrosis. The number of macrophages producing TNFα was significantly higher in the liver of IDO knockout animals, leading to a rise in the level of TNFα accompanied by the elevated expression of fibrogenic factors as compared to WT animals. On the grounds that IDO activation leads to a decrease in available Trp with the simultaneous production of kynurenine metabolites and that Trp is cardinal in the activation of NK and T cells, it was proposed that the elimination of IDO activity contributes to liver fibrosis by a dual mechanism: the inhibition of the enzyme results in sufficiently high Trp levels for lymphocyte activation and prevents the formation of kynurenine metabolites that suppress lymphocytes (32).

IDO seems to play an important role in intestinal immunity under normal circumstances as well. Harrington et al. reported a significant elevation of IgA and IgG in the gut and sera of IDO^−/−^ mice as compared to wild type animals (33). Antibiotic treatment of IDO^−/−^ animals led to a decrease in IgA and IgG levels indicating that the increased level of these Igs was a consequence of the lack of IDO modulatory effects on gut microbiota. Based on the observation that the elevated baseline Ig levels of IDO null animals could be corrected by antibiotic treatment, the authors proposed the involvement of IDO in a negative-feedback mechanism, which limits B lymphocyte responses to commensal microorganisms in the intestinal tract. This notion was supported by the finding that infection of IDO^−/−^ mice with a bacterial enteropathogen similar to the human pathogen *Escherichia coli, Citrobacter rodentium*, resulted in attenuated intestinal inflammatory responses. This was manifested in less oedema, cellular infiltration, epithelial damage and reduced intestinal colonization of the bacteria in IDO null animals as compared to WT (33). These beneficial effects were attributed to the elevated formation of natural secretory IgA, which facilitated the prevention of intestinal colocalization of the pathogen. It was hypothesized that IDO regulated gut microbiota by stimulating Ig production *via* the formation of cytotoxic kynurenine metabolites, as these kynurenines could inhibit the proliferation of the antibody producing B cells. Another mechanism by which the enzyme can affect B cell responses is by its ability to modulate T cell activity (33).

#### Effects of Genetic Modification of Indoleamine 2,3-Dioxygenase on Immune Responses to Cancer

A steadily growing body of data shows upregulated states of IDO in various cancer types making it a potent target for therapeutic approaches. To date, several chemical inhibitors of the enzyme have reached clinical trials, however, there are only a handful of those therapeutic approaches which attempt to modulate the enzyme function by genetic means [reviewed in (65)]. Besides post-translational modifications, the activity of the enzyme is also controlled at the transcriptional level (65), and in most interventions a decrease in the enzyme activity is desired, so genetic modulation seems feasible and exploring ways to achieve it is highly warranted. As upregulated IDO expression has been reported in various tumors (81), silencing the *IDO* gene could be an effective way for interfering immune escape in malignancies (Table 4). Report that *IDO* silencing by siRNA technology in cultured B16F10 melanoma cells diminished Trp catabolism and prevented apoptosis of T cells supports this notion. Transplantation of IDO inhibited tumor cells into mice resulted in the formation of smaller tumors. Moreover, *in vivo IDO*-siRNA treatment enabled the recovery of T cell responses, thus restoring host antitumor immunity, and silencing the gene also caused a delay in tumor onset (34). In melanoma mouse model, silencing injection of IDO specific shRNA, expressed from a plasmid in *Salmonella typhimurium*, attenuated tumor growth and led to a significant decrease in the number of lung metastases. In *Ido*-silenced animals massive tumor cell death was observed accompanied by polymorph nuclear neutrophil (PMN) infiltration in tumors. The production of excessive amounts of ROS led to the apoptosis of cancerous cells. Though it is likely that cytotoxic PMN recruitment is primarily to clear off S.t. cells, the production of ROS generates a microenvironment that is disadvantageous for tumor growth (35, 133). IDO silencing has been demonstrated to be effective in ovarian cancer as well (36). Injection of SKOV-3 human ovarian cancer cells with short hairpin RNA (shRNA) silenced IDO (SKOV-3/shIDO) into mice resulted in reduced tumor growth when compared to animals receiving IDO expressing cells. Simultaneously, peritoneal dissemination and ascites formation was inhibited and NK cell accumulation in the tumors was increased in SKOV-3/shIDO cell injected mice compared to those injected with tumor cells without IDO inhibition. *In vitro* studies revealed that in co-culture with NK cells, SKOV-3/shIDO cells displayed significantly decreased survival rates compared to those of non-IDO inhibited SKOV-3 cells, suggesting that IDO inhibition increases cancer cell sensitivity to NK cells (36).

In a recent study Wang et al. reported that radiotherapy (RT) treatment of patients with non-small cell lung carcinoma caused a decrease in KYN/Trp ratio, indicating diminished IDO activity, which was restored post-RT. They also reported a significant correlation between IDO activity and the clinical outcome of patients receiving RT. Those patients who had a higher KYN/Trp ratio prior to RT treatment showed significantly poorer survival than those with a lower KYN/Trp ratio. Similarly, there was correlation between greater KYN levels pre- and post-RT treatment and modest survival. These data suggested that RT induced favorable immune activity changes and IDO activity depended on the dose of implemented RT therapy. The authors hypothesized that defining the optimal dose of therapy is crucial in the modulation of IDO function, as a low dose would not be able to cause satisfactory immunomodulatory changes, whereas overdose can lead to detrimental impairment of the immune system (134).

The potential of enhancing cancer treatment efficacy by IDO function modulation was also demonstrated by Vareki et al. Anti-IDO shRNA transfected A549 human lung adenocarcinoma cells exhibit enhanced sensitivity to anti-cancer treatment. Genetic depletion of IDO sensitized the cells to the NAD+ inhibitor FK866, base excision repair (BER) inhibitor methoxyamine (MX), the folate anti-metabolite pemetrexed and the nucleoside analog gemcitabine—the latter two are already approved anticancer drugs. Simultaneous downregulation of IDO and thymidylate synthase (TS), a rate-limiting and key enzyme of DNA repair, led to the sensitization of the cells to 5FUdR as well. These results demonstrate the potential of genetic inhibition of IDO in combination with chemotherapy in cancer treatment (37).

Thus far, IDO targeting in cancer research and in therapeutic approaches involves mainly pharmaceutical enzyme inhibition. However, the use of IDO inhibitors has limitations [reviewed in (135)], underpinning the importance of genetic interventions, both alone and in combination therapy. Most of the known IDO inhibitors are analogs of the enzyme's natural substrate and act as competitive inhibitors of the enzyme. Thus, in order to exert the desired effect, these molecules either need to be used in concentrations at which they can compete with Trp in the target site, or need to show higher affinity for the enzyme than its own substrate. Trp analogs can also interfere with amino acid supply, thus misleading the cell, which in turn cannot give competent responses to changes in nutrient levels. A further limitation is that several of the applied drugs, such as 1-MT, Epacadostat, Norharmane, and Navoximod are AHR activators. This calls for specific attention, since there are data on AHR ligands possessing pro-carcinogenic effects [reviewed in (135)], though results are conflicting in regard of this question and further investigation is needed in order to clarify this issue. On the other hand, it must be emphasized here that similarly to the disadvantages and concerns regarding pharmaceutical enzyme inhibition, genetic modifications also carry dangers and raise several questions [reviewed in (136)]. There are concerns regarding the use of both viral and non-viral vectors and the off-target effects. Integration of the transfected genetic material into unwanted sites might evoke unwanted, potentially fatal immune responses for the host. While choosing sides between the two therapeutic approaches at present is hardly possible, in light of the progress of drug design and gene delivery techniques, it is likely that IDO targeting in either way or in combination will enter into the regiment of treatments of important malignancies.

### Tryptophan-Dioxygenase

Similarly to IDO, the effect of the functional ortholog enzyme, TDO, expression was studied on the immune response to tumors in animal models. Pilotte compared tumor rejection rate observed upon injecting TDO expressing and TDO non-expressing P815 tumor cells into the peritoneal cavity of mice (38). Though both cell lines produced tumors, the growth of tumors resulting from cells not expressing TDO was slower than those originating from cells which expressed TDO. TDO expression led to a decrease in T lymphocyte proliferation in the tumor microenvironment, indicated by the fewer cytolytic T lymphocytes (CTL) detected in the peritoneal cavity of animals. They concluded that TDO inhibition promotes tumor rejection. Furthermore, they concluded that inhibition of IDO and TDO might have synergestic effects in improving host response to tumors. Interestingly, pharmacological inhibition of TDO potentiated tumor-rejecting ability (38). These findings raise the possibility of targeting the TDO enzyme in an anti-tumor therapy.

While inhibition of T lymphocytes resulting from TDO expression can be detrimental for anti-tumor activity, the activity of the enzyme can be beneficial in the fight against infectious diseases. TDO expressing HeLa cells were found to exert antiparasitic, antiviral and antibacterial effects, as these cells were found to be able to inhibit the growth of *Toxoplasma gondii, Herpes simplex virus* and *Staphylococcus aureus* after tetracycline stimulation. Similarly to the finding of diminished T lymphocyte proliferation in the tumor microenvironment upon TDO expression, the presence of the enzyme resulted in the restriction of T cell proliferation in cells pre-treated with anti-CD3 mitogenic antibody. Furthermore, supernatant obtained from TDO expressing cells was capable of inhibiting allogeneic T cell responses. Both the antimicrobial and T cell proliferation inhibitory effects of TDO expressing cells were blocked by administering exogenous Trp, suggesting that the mechanism by which these effects are achieved is due to the decreased level of Trp available because of its metabolization by TDO (40).

A recent study of Elbers et al. revealed that hypoxia significantly impaired both antibacterial, antiparasitic and immunoregulatory properties of TDO. Investigation of TDO expression and enzymatic activity in HeLa cells engineered to express the enzyme and murine liver homogenates revealed that under low oxygen conditions, though the expression was not affected, the enzymatic activity of the protein was significantly reduced. In line with this, under hypoxic conditions, the growth of *Enterococcus faecalis* and *Staphylococcus aureus* was no longer inhibited, and T cell proliferation was restricted. Considering that hypoxia can often be observed in tumoral tissues and that infected tissues often exhibit low oxygen levels, the loss of normoxia could be a key factor in the loss of appropriate immune responses against pathogens and tumors (137).

Genetic inhibition of TDO was also investigated in EAE model of MS. Though TDO deficiency had no impact on leukocyte infiltration in the CNS, nor on the rate of demyelination, disease activity or degradation of the optic nerve, it had protective effects against neuronal loss in the spinal cord. This discrepancy could be explained either by the different sensitivities of these areas and/or by the diverse expression of TDO in separate brain areas (39).

As TDO is expressed in the liver, and some corticoids which are widely used in immunosuppressive therapy following transplant induce its expression (93, 138), it follows that TDO modulation might be exploited in allogeneic liver transplant protection. Reduction of locally available Trp in the liver can diminish T cell responses (40), while simultaneous production of kynurenines can promote the development of suppressive, rather than effector dendritic cells, thus further inhibiting T cell responses (138). At present, however, there is no available data on findings on the effects of the genetic modulation of *TDO* on liver transplantation. Taking into account the reasons mentioned above and the positive effects of *IDO* modulation on allograft rejection, investigating the possibility of *TDO* use in this respect seems warranted.

### Kynurenine 3-Monooxygenase

The third enzyme of the KP which has been subject to studies concerning its immunomodulatory roles is KMO. In light of the position of KMO in the hierarchy of KP enzymes, and that the induction of KMO is likely to shift the balance toward the production of neurotoxic and pro-inflammatory metabolites, targeting enzyme inhibition is a tempting approach for interfering in excessive inflammatory processes. Indeed, modulation of KYNA production by KMO inhibition has gained interest in the past decades and is a promising therapeutic approach for disease states linked to neurodegeneration, major depression, cancer, and immunological abnormalities (139). Besides utilizing specific KMO inhibitors, *Kmo* knockout animals are also efficient tools for investigating the effects of the lack of the enzyme (Table 5).

Giorgini et al. generated *Kmo*^−/−^ mice and investigated the levels of different KP metabolites in the brain, liver, and plasma of the animals. The levels of Trp and NAD^+^ tended to be only slightly decreased in these tissues. The marginal decrease in Trp level suggests that KMO inhibition has only a slight influence on upstream KP enzymes such as IDO and TDO. The findings that practically no difference in NAD^+^ levels was found supports the assumption that alternative NAD generating mechanisms are able to produce the necessary amount of metabolite when the KP is inhibited. The levels of KYN, AA, and KYNA were elevated, with a more striking increase in the level of the latter in the periphery than in the brain. Interestingly, though the levels of the product of the enzyme, 3-HK, were significantly decreased both in the periphery and the brain, the metabolite was still detectable. This observation suggests that in KMO^−/−^ animals other enzyme isoform(s) are capable of producing the metabolite in small quantities (139). Noteworthy was the difference between levels of QUIN in the periphery and in the CNS of *Kmo* null animals. A significant decrease in the level of this excitotoxic metabolite was detected both in the liver and plasma of KMO^−/−^animals, but there was only a moderate decrease in the brain. Based on these findings the authors concluded that peripheral inhibition of KMO might be sufficient for neuroprotection, as targeted inhibition of KMO in the CNS would not result in a more prominent decrease in the levels of QUIN (139). Though the decrease in the amount of 3-HK and QUIN accompanied by the increase of KYNA levels in the CNS could shift neurotoxicity toward neuroprotection, excessive elevation of KYNA holds plenty of danger (140). There is a growing body of evidence on the association of exaggerated KYNA levels and impairment in cognitive functions (101, 141–143).

Genetic modulation of KMO activity as a possible therapeutic approach for intervening infections was investigated by Kubo et al. in an EMCV induced mice model of viral myocarditis (41). They found that the survival rate of KMO^−/−^ mice was significantly higher compared to WT animals. This was accompanied by a significant decrease in the cellular infiltration of macrophages and neutrophils in the heart tissue of knockout animals. Moreover, the number of EMCV infected cells was significantly reduced in KMO^−/−^ specimens in parallel with lower levels of EMCV genomic RNA. Viral infection upregulated the expression of *Kmo* as significantly higher *Kmo* mRNA levels were detected in KMO^+/+^ animals after EMCV infection. These findings support the notion that links upregulated enzyme expression to higher mortality upon viral infection. Differences between WT and knockout animals were detected not only in the heart tissue but also in the periphery. In the serum of KMO^−/−^mice lower chemokine and cytokine levels, while higher levels of KYN and KYNA were detectable compared to WT animals (41). KYN has been shown to inhibit T and NK cell proliferation, and, *via* the generation of ROS, induces the apoptosis of NK cells (144–146). KYNA also exerts immune modulating effects by restricting TNF production of macrophages *via* G protein-coupled receptor GPR35 activation (147). Based on the anti-inflammatory effects of KYN and KYNA, elevated levels of these metabolites are proposed to be key factors in decreased inflammatory responses seen in KMO^−/−^ animals. Thus, genetic inhibition of the enzyme is expected to exert beneficial effects by preventing excessive cytokine and chemokine production and decreasing the recruitment of cells of the immune system (41).

In a mouse model of autoimmune gastritis, siRNA mediated gene silencing of *Kmo* led to the exacerbation of the disease. A self-regulatory mechanism was proposed, whereby the expression of *Kmo* ensures kynurenine catabolism, therefore reducing the amount of available AHR agonist kynurenine, thus lessening the formation of Th17 cells and pro-inflammatory IL-17 production. Accordingly, the inhibition of the enzyme exacerbated inflammatory processes *via* promoting the formation of Th17 cells (42).

Changes in KMO function have been reported in diseases linked to chronic inflammation as well. The expression of *KMO* in podocytes was found to be decreased in a diabetic environment, both in human and mouse kidneys (43). Genetic inhibition of the enzyme under diabetic conditions in mice and zebrafish resulted in proteinuria, a condition often related to diabetes. Serum kynurenine metabolite levels in these animals were changed showing an increase in the levels of KYNA and KYN parallel with a decrease in the level of AA, suggesting a shift in the KP. Depletion of NAD^+^ was found to have a negative effect on insulin sensitivity and also on the proper functioning of podocytes. Based on these findings, decreased expression and/or genetic inhibition of KMO leading to decreased production of NAD^+^ was proposed to contribute to diabetes related proteinuria (43).

In a recent study of Zheng et al., genetic deletion of KMO was found to be protective against ischemia-reperfusion injury (IRI) induced acute kidney injury (AKI). KMO^−/−^ mice showed better kidney function and significantly diminished tubular necrosis in the kidney tissue than KMO^+/+^ animals. Similarly, significantly less neutrophil granulocyte infiltration was measured in the renal tissue upon KMO deficiency. Following IRI, the levels of Trp were significantly decreased in both KMO^−/−^ and KMO^+/+^ groups compared to animals without IRI. IRI also led to increased levels of the protective KYNA in KMO^−/−^ mice. The plasma level of 3-HK was elevated followed by IRI in KMO expressing animals, however, such changes were not observed in KMO deficient animals. Considering the ROS generating, thus potentially pro-apoptotic properties of 3-HK, it could be concluded that genetic ablation of KMO, followed by decreased production of 3-HK, carry potential for preventing AKI after IRI (44).

Despite that these findings suggest promising results upon *KMO* ablation, inhibition of the enzyme might hold disadvantages as well. Recently Badawy has proposed, that QUIN is the most potent immunosuppressant KP metabolite, and it plays antagonistic role with the anti-inflammatory KNYA (148). Though downregulation of KMO might be beneficial in regards to elevated anti-inflammatory KYNA production, this would be accompanied by a diminished amount of the immunosuppressant QUIN. This situation clearly represents the complexity and delicate balance of the pathway and its metabolites, and raises further difficulties to be overcome.

## Associations Between Gene Variations Leading to Changes in KP Enzyme Function and Human Diseases

With the advent of new generation sequencing, a growing body of data is accumulating on polymorphisms in the human genome. It is, however, a great challenge to establish causality between genomic changes and the appearance and/or progression of specific diseases, and associations between genetic variants with disease states remain mostly obscure. Variants of genes encoding KP enzymes have been found to occur in frequencies differing between patient groups of specific diseases and samples of healthy population (1). Association of *IDO* variants have been suggested with depression (48, 149, 150) and autoimmune diseases such as systemic sclerosis (151) and Crohn's disease (152). *TDO* variants are believed to be associated with hypertryptophanemia (153) and psychiatric disorders such as Tourette syndrome (154) and autism (155). *KMO* mutations were found to be related to psychiatric diseases such as schizophrenia (156–161), bipolar disorder (162), and postpartum depression (163), and also in multiple sclerosis (164). Variations of the *AADAT* gene encoding the KATII enzyme were found to be associated with bacterial meningitis (165–167), changes in *KYNU* are believed to be related to essential hypertension (168, 169) and xanthurenic aciduria (170). An SNP in the *HAAO* gene encoding 3-HAO was found to be associated to hypospadiasis by a so far unknown mechanism (171), and changes in *ACMSD* were proposed to be linked with Parkinson's disease (PD) (172).

Despite the numerous findings pointing to possible associations of specific SNPs with specific diseases, only in very few cases are known where a change in a gene sequence results in a change in the activity of an enzyme connected to the disease, thus indicating direct causal link between gene variant(s) and disorder(s). In the following part of this section we summarize findings of those naturally occurring variations in KP genes that disturb the activity of the encoded enzyme and thus are proposedly linked to human diseases in which immune functions are altered.

Several *IDO* gene variants -present in the population with differing frequencies-have been shown to impact enzyme function (173). Nonetheless, there are only a very limited number of studies on these variants, despite the great clinical relevance they might have in a better understanding the pathomechanisms of specific diseases.

Systemic sclerosis (SSc), a connective tissue disease of which a hallmark is autoimmunity, is indicated by the infiltration of circulating antibodies and activated T cells in the affected tissues (151). Underlying the role of altered immune response in the disease are findings of disequilibrium of the pro-inflammatory Th17 and regulatory T cell functions in patients with SSc (174). Considering the T cell modulatory effects of IDO, one can easily foresee the involvement of the enzyme in the disease. Tardito and colleagues investigated the occurrence of five *IDO* SNPs in SSc (151). Three of these nucleotide alterations are located in the coding region of the *IDO* gene, which all result in amino acid changes, the other two are in the intronic regions. The frequency of the 5 SNPs in the worldwide population vary between 1 and 22 percent, and each occur both in homo- and heterozygous forms (based on data of www.ensembl.org). In the case of four out of the five SNPs involved in this study there were no observable frequency differences in their appearance among groups of SSc and control samples.

A comparison of the frequency of SNP rs7820268 between a group of SSc patients and a matching control group of healthy controls revealed significant difference (151). Rs7820268 is a change of a C to T within intron 5 of the *IDO* gene. The minor allele is present in 22 percent of the population worldwide with 37 percent as the highest population minor allele frequency (based on data of www.ensembl.org). The frequency of T allele was significantly higher among patients compared to healthy controls, and similarly higher were the frequencies of both genotypes carrying the T allele (TT and TC). By comparing the suppression activity of T regulatory cells of patients with at least one T allele to those homozygous for the C allele, it was shown that CD8+ Treg suppression activity was impaired in individuals carrying the minor (T) allele. The authors concluded that the rs7820268 *IDO* SNP possibly affects *IDO* expression and/or activity in a certain type of immune cells, e.g., DCs, which, *via* cell-to-cell crosstalk affect the suppressive function of CD8+ Treg cells leading to the evolution of autoimmune processes (151).

Association of *IDO* gene variants with another autoimmune disease, Crohn's disease (CD), has been reported by Lee et al. (152). CD is a chronic inflammatory bowel disease, which includes the involvement of the gastrointestinal tract and often several extraintestinal manifestations, further worsening the phenotype. In CD the expression of *IDO1* was found to be upregulated, and the activity of the enzyme was found to be in positive correlation with disease severity and inflammatory markers such as erythrocyte sedimentation rate and C-reactive protein (175). A comparison of the frequencies of 6 different *IDO1* variants among CD patients and healthy controls did not reveal significant differences, nevertheless the higher frequencies of three of the investigated variants were found to be associated with the severity of the disease (152). Both rs35059413 and rs35099072 are a C to T nucleotide change (indicated in reverse orientation) resulting in a Ala to Thr and Arg to His amino acid change in the 4th and 77th amino acid position of the protein, respectively (based on data of www.ensembl.org). The third variant is a C to A nucleotide change in exon 7. Patients carrying any of these three coding *IDO1* gene variants were more likely to show extraintestinal manifestations such as arthritis, uveitis, and perianal disease, indicators of CD severity. Serum KYN/Trp ratio, that is an indicator of *IDO* activity, revealed a decrease in enzyme activity in patients possessing *IDO1* SNPs. These findings strongly support the existence of an association between a more severe disease phenotype and a decrease in IDO1 activity which is likely to originate from mutations of the *IDO* gene (152).

Investigation of genetic variants of another KP gene, *AADAT*, that encodes KATII, revealed that in a SNP (rs1480544), a C to T change in the intronic region of the gene might be associated with bacterial meningitis (BM) (165). The minor allele was found to be more frequent among patients suffering from the disease than in healthy volunteers. The TT genotype was found to be accompanied by a decrease in several inflammatory markers in the blood, such as TNFα, IL1β, and IL6, and a diminished immune cell number in the cerebrospinal fluid (CSF) (165). These findings led to the hypothesis that this genetic variant could affect the course of the infection by influencing the recruitment of immune cells at the site of infection and the production of inflammation-related cytokines. Further studies focusing on this genetic variant revealed that in individuals with the TT genotype an increased level of KYNA and an elevation in the level of an anti-inflammatory cytokine, IL-10, were observed compared to those carrying the CC genotype (166). This led to the conclusion that BM patients bearing this *AADAT* SNP might expect a better disease outcome due to a less excessive inflammatory response. As the SNP is located in a putative exonic silencer, it is hypothesized that it leads to enhanced *AADAT* mRNA production and consequently to an increase in the amount of protein produced (166, 176). As a consequence, production of the neuroprotective and antioxidant KYNA is increased in carriers of the SNP, further alleviating neuronal damage caused by infection (166).

## Conclusions

In light of the large number of metabolites that are able to evoke neurotoxic vs. neuroprotectant, ROS generating vs. antioxidant, pro- vs. anti-inflammatory effects, it is no wonder that alterations of the KP have been linked to disease states. Indeed, altered kynurenine production has been shown in several neurodegenerative (177–179), psychiatric (180), and inflammatory diseases (181). Several of these ailments are characterized by altered immune functions, primarily as the result of altered levels of specific metabolites resulting from a change in the activity of key enzymes of the pathway such as IDO, TDO, or KMO. Interfering in the pathway in order to restore the imbalances of kynurenine metabolites could be a feasible way of ameliorating symptoms and reducing the progression of disease states. Results obtained using animal models indicate that frequently, though not exclusively, specific enzyme activity at either normal or increased levels could be the culprit behind unwanted immune reactions. On the other hand, this might offer possibilities to restore normal function by downregulating specific genes. The emerging technique of genetic therapy is a promising therapeutic approach that has been or is currently attempted in a regimen of diseases: e.g., neurological disorders such as Huntington's disease [IONIS-HTTRx; ClinicalTrials.govidentifier: NCT02519036; (182)] and spinal muscular atrophy (SMA) (140), immunological diseases as severe combined immune deficiency due to adenosine-deaminase deficiency [ADA-SCID; (183)], cardiovascular diseases (184) and malignant diseases like acute lymphoblastic leukemia (185) or non-Hodgkin lymphoma (186). In most cases genetic therapy is viewed as a technology by which entire genes or gene segments are inserted or removed to/from the genome. However, an effective genetic approach could be selective inhibition of the expression of a specific gene at a given tissue type and/or in a specific time frame. Results from animal models suggest that this could be a possibility in the cases of several diseases in which an overreaction of the immune response might be ameliorated by inhibiting KP enzyme activity locally and temporarily.

Regarding the modulation of immune function *via* the KP by genetic interventions, the first and rate limiting enzyme, IDO has been in the focus of research. Most of these studies use animal models, primarily mice knockouts. Several studies, on the other hand, were performed by modifying KP enzyme expression in animal or human cells in tissue culture. Inhibition of the enzyme at a genetic level was found to be beneficial in animal models of bacterial (2) and viral (3, 4) infections, immune modulation following organ transplant (19, 20) and diseases in which chronic inflammation plays a crucial role, such as DR (27), atherosclerosis (28), AAA (29, 30) and metabolic changes linked to obesity (31). Inhibition of the enzyme is also a promising way of interfering in tumor mediated immunological changes, thus restoring anti-tumor immunity (33, 34, 36, 45, 46). Genetic inhibition of a functional ortholog of the enzyme, TDO, was also found to be beneficial in the combat against tumors (38). On the other hand, cell culture studies also revealed antimicrobial effects of enhanced expression of the enzyme (40). Genetic modulation of another KP enzyme, KMO has also proven to be two sided: inhibition was found to be protective against EMCV infection in a mouse model of viral myocarditis (41), however, lack of KMO was reported to lead to autoimmune disease exacerbation (42) and also caused malfunction of podocytes in the kidney and consequent proteinuria (43).

Genome wide association studies have proposed several genetic variants of genes encoding KP enzymes to be associated with human diseases. However, there are only a handful of studies on the effects of these genetic changes on enzyme function. Thus, establishing cause-case relations between specific SNPs and disease development requires a great amount of further work. Nonetheless, findings obtained from genetically modified animal studies suggest that intervention in the KP by genetic modulation might be a promising therapeutic approach. Thus, research aimed at uncovering the effects of naturally occurring gene variations on the expression and function of the encoded enzymes is highly warranted, as results of these studies combined with preclinical findings can help in the identification of novel therapeutic targets and in the development of suitable therapeutic approaches.

## Author Contributions

LV and FB contributed conception and design of the manuscript. FB did data collection and wrote the manuscript in consultation with LV. LV contributed to manuscript revision and to the refinement of the final manuscript. All authors read and approved the submitted version.

### Conflict of Interest

The authors declare that the research was conducted in the absence of any commercial or financial relationships that could be construed as a potential conflict of interest.

## Abbreviations

KP: kynurenine pathway
IDO: indoleamine 2,3-dioxygenase (protein)
*IDO*: indoleamine 2,3-dioxygenase (gene in human)
*Ido*, indoleamine: 2,3-dioxygenase (murine gene)
TDO: tryptophan 2,3-dioxygenase (protein)
*Tdo*: tryptophan 2,3-dioxygenase (murine gene)
*TDO*: tryptophan 2,3-dioxygenase (gene in human)
KMO: kynurenine 3-monooxygenase (protein)
*KMO*: kynurenine 3-monooxygenase (gene in human)
*Kmo*: kynurenine 3-monooxygenase (murine gene)
CNS: central nervous system
L-KYN: L-kynurenine
KYNA: kynurenic acid
KATI-IV: kynurenine aminotransferases
NMDAR: N-methyl-D-aspartate receptor
α7nAChRs: nicotinic acetylcholine receptors
KYNU: kynureninase
AA: anthranilic acid
3-HK: 3-hydroxy kynurenine
XA: xanthurenic acid
3-HAA: 3-hydroxyanthranilic acid
ACMS: 2-amino-3-carboxymuconate-semialdehyde
3-HAO: 3-hydroxyanthranilate 3,4-dioxygenase
PIC: picolinic acid
ACMSD: aminocarboxymuconate-semialdehyde-decarboxylase
QUIN: quinolinic acid
ISRE: IFN stimulated response element
GAS: gamma-activated sequences
DRE: dendritic cell response element
NF-κB: nuclear factor kappa-light-chain-enhancer of activated B-cells
TLRs: toll-like receptors
TGFBR: transforming growth factor beta receptor
AHR: aryl hydrocarbon receptor
IFNBR: interferon beta receptor
IFNGR: interferon gamma receptor
TNFR: tumor necrosis factor receptor
SOCS3: suppressor of cytokine signaling 3
DC: dendritic cell
IFN: interferon
LPS: lipopolysaccharide
IL: interleukines
TNF: tumor necrosis factor
mTOR: mammalian target of rapamycin
NPC: nasopharyngeal carcinoma
AML: acute myeloid leukemia
AHR: aryl hydrocarbon receptor
ROS: reactive oxygen species
siRNA: small interfering RNA
IDO^−/−^: *IDO* knockout
UPEC: Uropathogenic *Escherichia coli*
MuLV: murine leukemia virus
NK cell: natural killer cell
pDC: plasmocytoid dendritic cell
WT: wild type
ECMV: encephalomyocarditis virus
sJIA: systemic juvenile idiopathic arthritis
sHLH: secondary hemophagocytic-lymphohistiocytosis
STING: Stimulator of Interferon Genes
DNP: DNA nanoparticle
EAE: experimental autoimmune encephalitis
MS: multiple sclerosis
MOG: myelin oligodendrocyte glycoprotein
c-diGMP: cyclic diguanylate monophosphate
CIA: collagen induced arthritis
RA: rheumatoid arthritis
AdIDO: adenoviral vector-mediated IDO gene delivery
NOD: non-obese diabetic
MRL*lpr/lpr*: Lupus-prone Murphy Roths large mice
SLE: systemic lupus erythematosus
MAS: macrophage activation syndrome
DR: diabetic retinopathy
AngII: angiotensin II
*ApoE*^−/−^: Apolipoprotein E knockout
NAD(P)H: nicotinamide adenine dinucleotide phosphate
AAA: abdominal aortic aneurysm
VSMC: vascular smooth muscle cell
*Ldlr*^−/−^: low density lipoprotein—receptor deficient
HFD: high fat diet
MMP2: matrix metallopeptidase 2
WAT: white adipose tissue
OGTT: oral glucose tolerance test
ITT: insulin tolerance test
IAA: indole-3 acetic acid
CCl4: carbon-tetrachloride
PMN: polymorph nuclear neutrophil
S.t.: *Salmonella typhimurium*
shRNA: short hairpin RNA
RT: radiotherapy
BER: base excision repair
MX: methoxyamine
TS: thymidylate synthase
IRI: ischemia-reperfusion injury
AKI: acute kidney injury
CTL: cytolytic T lymphocyte
PD: Parkinson's disease
SSc: systemic sclerosis
CD: Crohn's disease
BM: bacterial meningitis
CSF: cerebro-spinal fluid.
